# An Experience of Video Based Training on Basic Life Support

**DOI:** 10.31729/jnma.3645

**Published:** 2018-08-31

**Authors:** Roshana Shrestha, Ashis Shrestha, Kabita Hada Batajoo, Rashmi Thapa, Samita Acharya, Sumana Bajracharya, Sanij Singh

**Affiliations:** 1 Department of General Practice and Emergency Medicine, Kathmandu University School of Medical Sciences, Dhulikhel, Kavre, Nepal; 2 Department of General Practice and Emergency Medicine, Patan Academy of Health Sciences, Patan, Nepal; 3 Department of General Practice and Emergency Medicine, KIST Medical College and Teaching Hospital, Imadol, Nepal; 4 Department of General Practice and Emergency Medicine, Kathmandu Medical college and Teaching Hospital, Nepal; 5 Department of Emergency Medicine, Nepal Mediciti Hospital, Kathmandu, Nepal

**Keywords:** *basic life support*, *cardiac arrest*, *cardiopulmonary resuscitation*, *simulation*, *video-based training*

## Abstract

**Introduction:**

Basic life support is foundation to save lives. In contrast to the developed countries, there is still no national standard BLS training module in Nepal. Basic life support training is being provided by various institutions but lack in consistency and coordination. The Nepal basic life support Course is the video based training in Nepali language with reference to recent advances which was intended for all health care personnel of Nepal in urban as well as rural setting. We aimed to describe the features of this video based training module in local language, to analyse the differences of knowledge before and after the training and to find out the participants perception and satisfaction with this course.

**Methods:**

This is a descriptive cross-sectional study based on data of trainings conducted over the study period. Ethical approval was taken. The post-test score was recorded and compared with the occupational using ANOVA. On the spot and delayed feedbacks from the participants were collected voluntarily and summarized.

**Results:**

Total of 576 participants (435 clinical doctors, 92 nurses/paramedics, 18 non-clinical doctors and 41 intern doctors) successfully completed the training. The difference in post test scores (mean = 12.9+1.8) among the different occupational background was not significant (P=0.159). The feedbacks from the participants were mostly positive and encouraging.

**Conclusions:**

The knowledge of basic life support improved significantly irrespective of the occupation of the participants. A universal, nationwide video based training module in Nepali language should be developed focusing all health care personnel of urban as well rural Nepal.

## INTRODUCTION

The healthcare professionals of any background must know the BLS skills which are fundamental to save lives.^1,2^ In the developed countries, BLS training is mandatory for all health care professionals and is standard, universal and updated,^3^ however there is still no universal mandatory training for healthcare personnel in our country. There are many academic institutions providing the BLS training currently but in silos.

The Nepal BLS for Healthcare Providers Course is the video based training in Nepali language with reference to recent American Heart Association (AHA) guidelines^4^ which was designed for all health care personnel of Nepal in urban as well as rural setting to be able to provide basic life support in a safe, timely and effective manner.

This descriptive study describes the training module and analyses the differences in post-test score among different occupational group. This study also aims to evaluate the feedbacks given by participants upon completion of the training.

## METHODS

In this descriptive cross-sectional study, the data of trainings from November 2014 - May 2018 was analysed. The trainings were conducted at various institutions of Nepal (Nick Simons Institute, Kist Medical College, Manmohan Memorial Medical College, Mediciti Hopital, Star Hopital, Kirtipur Hopital and National Health Training Centre) focusing the health care personnel of different professional background. They were categorized into clinical doctors (doctors working in clinical settings), non-clinical doctors (doctors working in non-clinical settings- dental, ayurveda, basic sciences), nurses/paramedics and interns. The identity of the participants was not disclosed in the analysis of the data. The feedback forms didn't contain the name of the participants, thus the confidentiality of the participants was maintained. The ethical approval was taken. Data of all the participants who completed the training during this period was included.

Two pilot training sessions were conducted in initial phase to test the training video, student manual and the testing materials. Appropriate remediation was undertaken after the feedback from these sessions. There were 15 multiple choice questions (MCQ) which were taken from a previously validated study^5^ with permission and modified according to the 2015 AHA guidelines.^4^ The questionnaire underwent expert panel consultation and then was validated in the pilot trainings. The questionnaire contains vignette based multiple choice questionnaire with one right option on knowledge and skills of adult BLS, infant BLS and choking. The written pre-tests were not mandatory and depended upon the wish of the course coordinator before the course. Participants had to secure 80% in the post tests to pass the exam and to be certified. If the goal was unreachable in first attempt remediation was done maximum for two times till satisfactory performance. Structured skill test sheets for adult and infant BLS was developed and tested in the pilot study which was used to check the participant's performance in the BLS skills. The box was ticked only if the participant correctly performed the skill. If they did not perform a specific skill correctly, it was noted on the sheet. At the end of the test the student was informed about the skills they needed to practice further. They had opportunity to redo the test at the end of the course during “remediation”.

At the end of the all training sessions a voluntary “on the spot feedback” on participants' satisfaction was obtained with a predesigned form for qualitative data collection. This form was divided into five sections-general feedbacks, feedbacks on adult BLS, infant BLS, choking using five point Likert's scale and open comments.

An online feedback form “delayed feedback” was developed by the authors and disseminated on May 2018 to all the participants who had completed the training via email to explore their perception about the course. The participation was voluntary and anonymous. The form contained demographic date: age, occupation, duration of work experience, date of Nepal BLS training, frequency of involvement in resuscitation of patients; five tier Likert scale to explore the perception about video based training in local language using 7 items; followed by their open opinion about future courses and refreshers training.

The demography, occupation, pre-test/post-test scores were recorded in Microsoft Office Excel. Data analysis was performed using the statistical software SPSS Version 23. The difference in post test scores among the different occupational background was analysed with ANOVA. P<0.05 was considered as statistically significant. The feedbacks were recorded, quantitative data were analysed in proportion. Qualitative data were classified based on themes and analysed.

## RESULTS

There were total 26 training sessions at different institutions and hospitals of Nepal with total of 576 participants during the three and half year period. Among those participants 425 (73.8%) were clinical doctors, 92 (16%) were nurses and paramedics, 18 (3.1%) were non-clinical doctors and 41 (7.1%) were intern doctors.

Among the total 576 participants the mean post-test was 12.9±1.8. The difference in post test scores among the different occupational background was not significant when analysing with ANOVA (P = 0.159) (Table 1).

**Table 1:** Post- test results and occupation of the participants.

Test	Occupation	n (%)	Mean score	Standard deviation	P (ANOVA)
Post-test	Clinical doctors	425/576 (73.8)	12.0	1.9	0.159
	Nurses/paramedics	92/576 (16)	13.1	1.3	
	Nonclinical doctors	18/576 (3.1)	13.2	1.4	
	Interns	41/576 (7.1)	13.4	0.9	
	Total	576	12.9	1.8	

As per participants performance the box in the structured skill testing sheet was ticked. Among the 576 participants 46 (7.9%) required remediation as some critical steps were missed in first attempt. Following further practice and remediation all of them completed the skill testing successfully.

**Figure 1.**
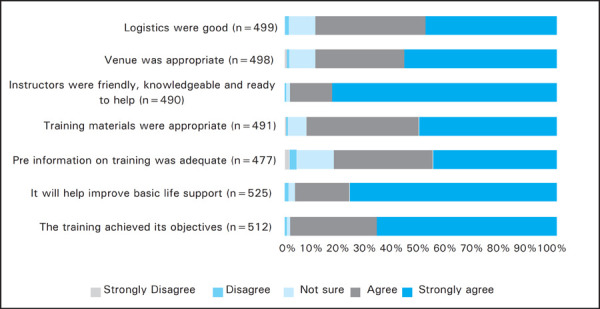
General feedback from the participants.

**Figure 2.**
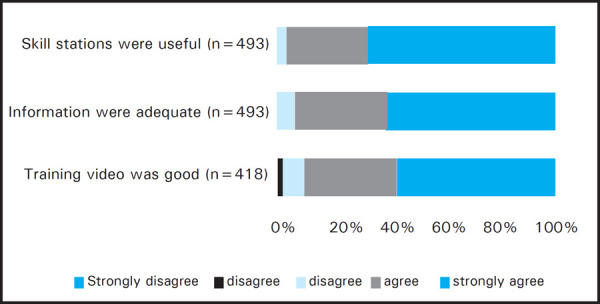
Adult BLS feedback.

**Figure 3.**
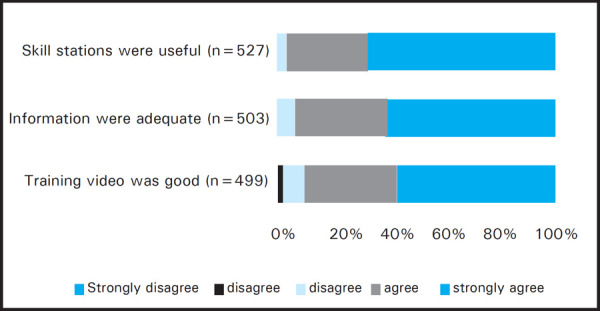
Infant BLS Feedback.

**Figure 4.**
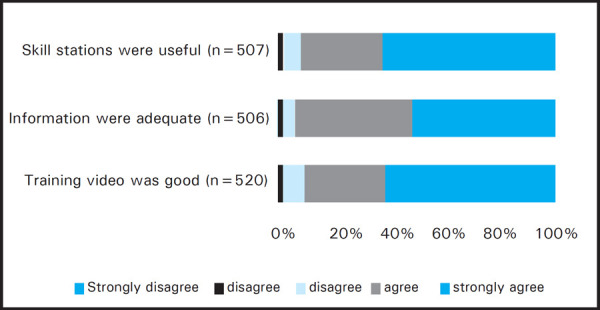
Choking Feedback.

As the feedback process was voluntary, the number of responses varied in different sections of the feedback form. Among the total responses (512 out of 576), 339 (97%) participants strongly agreed or agreed that the objectives of the training were met (Figure 1). About 510 out of 525 (97.2%) participants agreed that the training would improve their BLS skills. The videos for adult BLS, child/infant BLS and choking were rated strongly agreed and agreed in combination as 436 out of 480 (90%) responders, 455 out of 499 (90%) responders and 478 out of 520 (92%) responders respectively (Figure 2,3,4). Most unsatisfactory result was obtained for the pre-information of the training (negative- 22/477 (4%); neutral-64/477 (13%).

Some of the open comments in the feedback form by the participants were categorized into positive and constructive. One of their comments from the participant- “I found this training is of high standard and it is very useful in enhancing knowledge about resuscitation and can be applied in practical life”. The specific areas of satisfaction were as follows: videos (289), simulation skill stations (223), training materials (89), trainers' knowledge and friendliness (132), planning of training (69), and skill testing (48). Specific positive phrases used for video were, “impressive”; “clear”, “interesting”; “informative”, “well explained”, “innovative” and “effective”: One of the participants comment -“The training is fruitful to each and every medical personnel specially the presentation style of the training video in Nepali language and simulation is the most effective part.” Constructive feedbacks for video were “needs to be updated”, “needs improvement in sound” and “more practical”. There were many future recommendations in regards to the number of the AED (152). They wanted more number of AED to practice and more skill stations on AED. “In our hospital settings across the country, very few centers have AED facilities, and in clinical practice, it's replaced by conventional defibrillator, demonstrating it would be difficult but adding a short introduction about conventional defibrillator (types available, how to use etc.) would help the BLS participants a lot.” There were contrary statements in regards to the duration of the training. Some also expressed their wishes to attain other similar advanced training in resuscitation.

**Figure 5.**
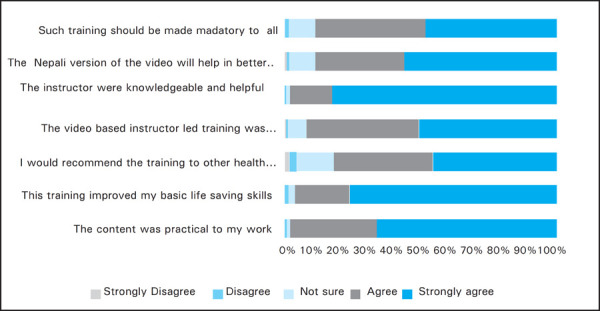
Delayed Feedback.

The response rate to the delayed online feedback was 106 (18.4%) till end of May 2018. Of the 106 responders, 89 (84%) were clinical doctors, 12 (11.3%) were nurses/paramedics and 5 (4.7%) nonclinical doctors. Out of them 58 (54.7%) were male. The duration of clinical experience was divided into <1 year, 1–3 years, 3–6 years and >6 years which comprised of 2 (1.9%), 74 (69.8%), 23 (21.7%) and 7 (6.6%) participants respectively. The details of the rating to the statements in Likert scale is summarized (Figure 5).

When the participants were inquired about any involvement in resuscitation after the Nepal BLS training, 28 (26.4%) of them had claimed they were frequently involved (>10 times per year), 38 (35.8%) were involved sometimes (5–10 per year), 35 (33%) occasionally (<5 times per year) and 5 (4.7%) were never involved. One hundred and four participants (98.1%) of the participants were interested in taking such lifesaving skills in future. When the opinion about the interval of refreshers course was explored, 65 (61.3 %), 36 (34%), 3 (2.8%) and 2 (1.9%) of the participants stated that it should be done annually, every two years, five years and quarterly respectively. The phrases used to comment on the video based training was “good”, “effective”, “great”, “helpful”, “to the point”, “high standard”, “best”, “worth”, “sufficient”, “phenomenal”. Some participants (14) were concerned that the duration was short and advised that the duration be prolonged to at least 2 days with more practical skill stations and inclusion of neonatal life support and Advanced life support. Some (22) also commented on the need of refreshers course and dissemination of the course among all healthcare workers including those in rural areas. Few commented that the quality of video (2) and audio (5) should be improved with updated content (9).

## DISCUSSION

This is the first video based BLS training in local language in Nepal. Google, Google scholar and PubMed based search did not reveal citation of any other video based BLS training in Nepal. The training is conducted at various health institutions targeting different health care professionals in Nepal from 2014. Till date 47 participants of different background including general practitioner, surgeon, orthopaedic surgeon, anaesthesiologist and paramedics have received the training for teachers (TOT) course and are instructors for Nepal BLS. This video was initially based on AHA guidelines 2010 and was subsequently updated to 2015 version^4^ as new guidelines were introduced. The

AHA certified BLS is also available in Nepal but may not be affordable, comprehensible and feasible in our part of world. One of the main features of this training module is the use of modern methods of teaching. The modification of the scenarios and dramas according to the local context makes it practical and feasible in real working scenarios in resource limited countries like ours. Up to present, several publications have highlighted and have addressed the current level of awareness and knowledge in this area among the health care professionals and have emphasized the deficiencies in basic life support skills in Nepal. ^5–7^

This video based, instructor-led course teaches the practical skills of both single-rescuer and team-based BLS skills for application in both in- and out-of-hospital settings and trains participants to promptly recognize arrest victim, give high-quality chest compressions, deliver appropriate ventilations, relieve choking and also introduces the use of automated external defibrillator (AED). Hundred and four participants (98%) recommended this training to other health care personnel in the delayed feedback. Ninety-three (87.6%) participants claimed that the content was practical to their work and 97 (91.4%) stated that it improved their BLS skills. Hundred and four participants (98%) gave positive response to the statement that such training should be made mandatory to all health care professionals in Nepal.

The video contains dramatized illustration of real life scenarios based on actual context to our part of world, demonstration of the various skills required for resuscitation and uses “practice as you watch” approach. The video based technique ensures consistency in future trainings irrespective of the instructor quality or mood in contrast to the lecture based trainings. The quality of the video was also rated high (90%, 92% and 90%) by the participants for adult, infant and choking sections of the video respectively. While analysing the open comments there were many positive feedbacks and encouraging words for the video-based training module (289). In the delayed feedback 96 (90.5%) of the participants thought that the video based instructor led training was superior to the traditional lecture based training. There are few literatures which state that video based learning is better than traditional classroom teaching with lectures.^8–10^ “Instructor led, hands on” class format has reinforced skills proficiency in this training. “Practice while watching” technique allowed Instructors to observe the participants, provide feedback and guide them to acquire the skills. This approach is also found to be efficient in other studies.^9^ Nepali language being officially and universally spoken all over Nepal, the message might be clearly delivered to Nepali health care personnel by using video based learning in local language. Ninety-four (88.6%) agreed positively that the Nepali version of the video will help better understanding for all health care personnel in the delayed feedback

Following the video based demonstration and “practice while watching” sessions the students were divided into small groups and real life time simulations were practiced to reinforce the skills learnt during the video session. Moreover, the importance of teamwork and coordination was also experienced by the participants in these simulation sessions. Simulation based medical education is a complex educational intervention and is well established as a practical, safe, structured and effective training strategy to train health care professionals in both technical and nontechnical skills which are known to be critical to effective teamwork.^11^ The adult, infant and choking skill stations were rated positive in 97%, 94.5% and 91% respectively. Positive comments about the simulation based skill stations were mentioned by 223 participants in the open comments and some of them wanted to increase the time and numbers of simulations. Written post-test was done to ensure participant acquired the knowledge of BLS. The written post-test score after the training was similar in each occupational group which demonstrates that the training improved the knowledge of the participants irrespective of their background occupations. While analysing the on the spot feedback, most of the participants agreed or strongly agreed to the statements given in the feedback form. Most of the participants (98.7%) gave positive rating for the statement that Instructors were friendly, knowledgeable and ready to help The reason for relatively constructive feedback was on response for the pre-information (negative- 4%; neutral- 13%) of the training could be that in some training the student's manual couldn't be distributed before the training due to various reasons. Most of the cardiac arrests in adults are due to ventricular fibrillation which is otherwise fatal within minutes though highly reversible with the rapid application of defibrillator.^12^ Recent developments in automated external defibrillator technology have provided a means of increasing the rate of prompt defibrillation after out-of-hospital cardiac arrest.^13,14^ Although AED is not widely available in Nepal, the course introduces and highlights the use of an AED so that its use will be fortified in future. In open comments there were 152 comments on AED. They wanted more practice time, demonstration and number of AED. Therefore the training in AED for future, such trainings are recommended.

This video based method of training was not compared to other conventional method of teaching so it cannot be proven from this study that video-based training is superior to the other methods. Moreover, the test was not repeated after some interval of the training to check for significant deterioration of knowledge with time.^14^ Due to anonymous feedback we couldn't categorise the feedback according to the background profession. The training is ongoing and this is just a cross-sectional study so generalization is not reliable. The response rate for delayed feedback was low (18%) and mostly clinical doctors and nurses/paramedics responded so the opinions can't be generalized to all cadres.

## CONCLUSIONS

This video based training in BLS in Nepali language significantly improved knowledge of BLS irrespective of the occupational background of the participants. It is recommended that a national, standard BLS training based on recent updates should be a core competency across all health care professional programs. Considering the feedbacks from the participants, video based training method can be considered a useful technique in such educational programs to ensure consistency and quality. Developing video based training module in local language may also increase the access to the BLS training as this should be adopted in a higher scale to cover all level of health care providers of Nepal including rural and urban areas to be able to provide BLS in a safe, timely and effective manner.
